# Distribution of cervical lesions in high-risk HPV (hr-HPV) positive women with ASC-US: a retrospective single-center study in China

**DOI:** 10.1186/s12985-020-01455-2

**Published:** 2020-11-23

**Authors:** Zhiling Wang, Ying Gu, Hui Wang, Junyu Chen, Yawen Zheng, Baoxia Cui, Xingsheng Yang

**Affiliations:** 1 Department of Obstetrics and Gynecology, Qilu Hospital of Shandong University, Wenhua West Road 44, Jinan, China; 2 Department of Obstetrics and Gynecology, Peking Union Medical College Hospital, Beijing, China

**Keywords:** Uterine cervical neoplasms, Human papillomavirus, Atypical squamous cells of undetermined significance, Cytology

## Abstract

**Background:**

To investigate distributions of cervical lesions and factors associated with the severity of the cervical lesions in high-risk HPV (hr-HPV) positive women with atypical squamous cells of undetermined significance (ASC-US) cytology.

**Methods:**

Clinical information of 250,000 women who underwent HPV and cytological test was collected from January 2012 to January 2019. The association between the severity of the cervical lesions and hr-HPV genotypes, hr-HPV viral load, and ages, were analyzed in hr-HPV-positive/ASC-US women.

**Results:**

3459 hr-HPV-positive/ASC-US women were enrolled in this study. Overall, 43.51% of women with ASC-US had normal histological results, 34.35% had high-grade squamous intraepithelial lesion (HSIL), and 1.30% had cervical cancer. The rate of HSIL or worse (HSIL+) in women with single HPV16 infection (63.09%) was the highest, followed by HPV33 (57.50%), HPV51 (36.11%), HPV58 (36.11%), HPV52 (28.28%), HPV18 (26.37%), HPV66 (19.35%), HPV39 (18.92%), HPV53 (15.00%), and HPV56 (8.51%). Detection rate of HSIL+ in low, intermediate and high viral-load groups were 15.87% (n = 30), 34.91% (n = 74) and 40.68% (n = 214) (Cochran-Armitage Trend test χ^2^ = 35.03, *p* < 0.0001). Compared with the 51–60-year-old group (21.65%), the women in ≤ 30 (40.52%), 31–40 (39.67%), and 41–50 (34.22%) year-old groups had significantly higher risk of HSIL+. The women in ≤ 51–60 (2.68%) and > 60 (3.41%) year-old groups were at increased risk for cervical cancer, compared with the ≤ 30-year-old group (0.61%).

**Conclusions:**

ASC-US women with HPV 16/18/33/51/52/58 single infection and multiple infections, as well as high HPV viral loads, have high risk of HSIL+.

## Background

Cervical cancer is the third most common cancer among women worldwide and is the second most common to breast cancer in Asia [1]. Approximately 90% of cervical cancer deaths occur in developing countries, which is higher than that of developed countries [2]. Persistent high-risk human papillomavirus (hr-HPV) infection is main cause of cervical lesions [3, 4]. At present, more than 200 genotypes of HPV have been isolated and the carcinogenicity of different HPV genotypes varies widely [5]. HPV16 causes 60% of cancers and 50% of precancerous lesions, however, HPV56 rarely causes cancer [6–8]. It takes 20–30 years from precancerous lesions to cervical cancer, and such long period provides doctors possibilities for intervention [9, 10]. Cervical cytology test and HPV test are the most common methods for cervical cancer screening.

Thinprep cytologic test (TCT) is a commonly used method for cytology test [11]. However, only 7–10% of ASC-US women was diagnosed as cervical intraepithelial neoplasia (CIN) 3, and there was very low probability of invasive carcinoma for ASC-US women [12].

There are three common HPV tests in China. The Cobas 4800 HPV test performs DNA extraction, Polymerase Chain Reaction amplification, and real time detection by an automated sample preparation, to detect 14 hr-HPV genotypes. The HPV GenoArray test simultaneous identification of 21 individual HPV genotypes and efficient detection of single or multiple HPV infection, which preforms DNA amplification and HybriBio’s proprietary flow through hybridization technique. Hybrid capture 2 test (HC2) is one of the most frequently applied test to detect the presence of any 13 hr-HPV types, which can provide a quantitation of the viral DNA load.

This study aimed to investigate the distribution of hr-HPV genotypes, the association between the severity of the cervical lesions and hr-HPV viral loads, and age-stratified prevalence of high-grade squamous intraepithelial lesion (HSIL) and cervical cancer, by analyzing of clinical information of HPV-positive/ASC-US women in China, which would help us find an appropriate triage of women with ASC-US cytology in the future.

## Methods

### Study population

All procedures in studies involving human participants were performed in accordance with ethical standards and approved by the Ethics Committee of Shandong University [2018 (054)].And the requirement for written informed consent was waived by the Ethics Committee of Shandong University.

This retrospective study included women who accepted TCT test, HPV test and colposcopy at Qilu Hospital of Shandong University from January 2012 to January 2019.

Inclusion criteria: (1) women with ASC-US cytology and hr-HPV infections; (2) women accepted colposcopic examination and underwent cervical biopsy under colposcopic guidance; (3) women with complete cervical cervix.

Exclusion criteria: (1) women with history of treatments to cervical lesions, such as cervical surgery, laser, freezing and medication, et al.; (2) women with malignant tumors such as ovarian cancer and endometrial cancer; (3) women with autoimmune diseases or receiving immunotherapy; (4) women with pregnancy; (5) women who had received the HPV vaccine; (7) women with smears of insufficient quality (with an absence of endocervical cells).

### Cytology test and HPV test

No vaginal washing, intravaginal medication or physical therapy for 3 days before the samples were collected. No sexual behavior within 24 h and women were in non-menstrual period when the samples were collected. Specimen collection, specimen preparations, and results of test are performed, according to the instructions of the manufacturers, respectively.

ThinPrep (Hologic Inc, Marlborough, Massachusetts, USA) preparations was used and cytological diagnosis was performed by experienced cytopathologists, according to the Bethesda system 2011 [13].

Women could choose one of three HPV tests:

1. HC2 (Qiagen, Gaithersburg, MD, USA): semi-quantitative detection of 13 hr-HPV types: 16, 18, 31, 33, 35, 39, 45, 51, 52, 56, 58, 59 and 68. Positive by the ratio relative-light-unit/cut-off (RLU/CO) was > 1.0 (equivalent to 1.0 pg HPV DNA/mL or 100 000 HPV copies/mL).
2. the Cobas 4800 System (Roche Diagnostics Corporation, Indianapolis, Indiana): Qualitative detection of HPV 16, HPV 18 and 12 hr-HPV (a pool of 12 other hr-HPV types: including HPV 31,33,35,39,45,51,52,56,58,59,66 and 68).
3. the HPV GenoArray test (Hybribio Biotechnology Ltd Corp, Chaozhou, Guangdong Province, China): qualitative detection of 15 kinds of hr-HPV types: 16, 18, 31, 33, 35, 39, 45, 51, 52, 53, 56, 58, 59, 66 and 68; 6 kinds of low risk HPV (lr- HPV) types: 6, 11, 42, 43, 44 and 81 (CP8304).

### Colposcopy and guided cervical biopsies

All women, enrolled in this study, underwent visual inspection with acetic acid and insulin inspection with Lugo's iodine (VILI) and colposcopy. If any of suspicious lesion were found among the above-mentioned examinations, multi-point biopsy was taken directed by colposcopy at the suspected lesion; otherwise, a random four-quadrant cervical biopsy was obtained at the squamocolumnar junction. Women who were not satisfied with colposcopy should underwent Endocervical Curettage, if necessary.

The histologic diagnosis was based on the consensus diagnosis of two experienced gynecologic pathologists. All gynecologic pathologists were blinded to results of TCT, HPV test and colposcopic examination. The results of biopsy reports include: Normal; low-grade squamous intraepithelial lesion (LSIL) (including CIN 1), high-grade squamous intraepithelial lesion (HSIL) (including CIN 2–3), and cervical cancer. HSIL or worse (HSIL+) includes HSIL and cervical cancer. The gold standard for this study was the histologic diagnosis.

### Role of the funding source

This work was supported by the foundation of National Natural Science Foundation of China and Science, Grant/Award Number: 81874105; National Clinical Research Center for Gynecological Oncology, Grant/Award Number: 2015BAI13B05; National Natural Science Foundation of China and Science, Grant/Award Number: 81372809; and National Key Research & Development Program of China, Grant/Award Number: 2016YFC1302903. The funding sources acknowledged the design of the study but did not influence the process of our study, including data collection, analysis, and interpretation and the reporting of results.

### Statistical analysis

Statistical analyses were performed using the SAS 9.4 (SAS Institute, Cary, NC) for windows. The categorical variables were expressed by a percentage (%). Cochran-Armitage Trend test was used to analyze the detection rate of HSIL+ in different viral load groups. The binary Logistic regression model was used to analyze the detection rate of HSIL+ and cervical cancer in different age groups. Results were considered statistically significant at *p* values < 0.05.

## Results

### Characteristics of the study population

**Fig. 1:**
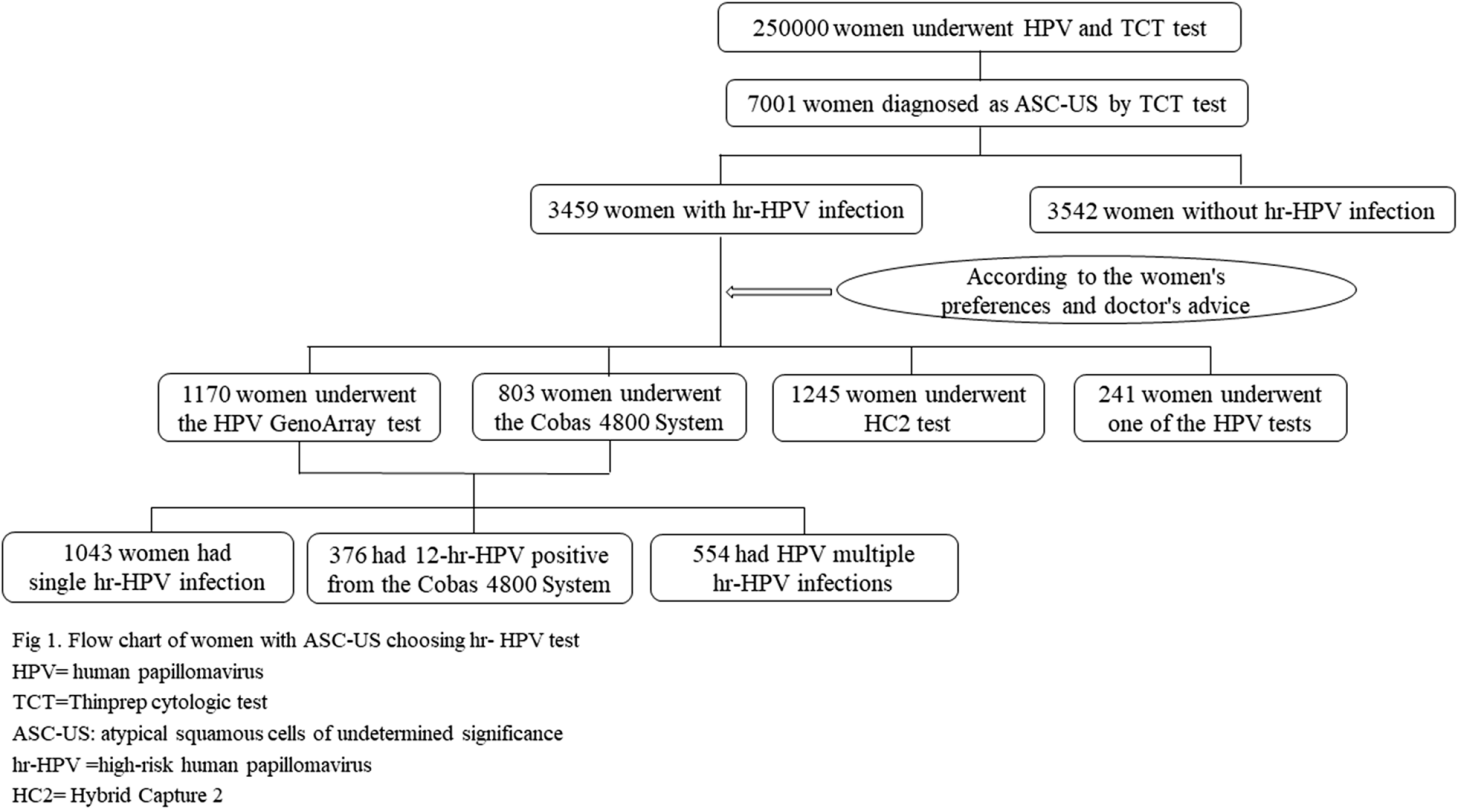
Flow chart of women with ASC-US choosing hr- HPV test. HPV = human papillomavirus, TCT = Thinprep cytologic test, ASC-US: atypical squamous cells of undetermined significance, hr-HPV = high-risk human papillomavirus, HC2 = hybrid capture 2

Clinical information of 250,000 women who underwent HPV and TCT test at Qilu Hospital of Shandong University from January 2012 to January 2019 was retrospectively collected and analyzed (Fig. 1). Among them, 7001 (2.80%) women were diagnosed as ASC-US by TCT test, including 3459 (49.41%) women with hr-HPV infection and 3542 (50.59%) women without hr-HPV infection. Finally, a total of 3,459 ASC-US women with hr-HPV infection were enrolled in this study finally.

A total of 1973 women chose the HPV typing test, identified the specific types of hr-HPV they were infected with, among whom 376 were 12 hr-HPV positive (from Cobas 4800 HPV test), 1,043 women had single HPV infection, and 554 women had multiple HPV infections. 1245 women were diagnosed with hr-HPV positive by HC2 test. The remaining hr-HPV positive 241 women who lost their results of HPV test, were classified as ANY group in this study. (We did not know the specific genotypes, nor did we know whether it is a single infection or multiple infections. We only knew that they were hr-HPV positive by their medical records during this study period.)

The average age of women was 39.86 ± 9.94 years (range: 25–80 years). The median number of pregnancies was 3 (range: 0–14) and the median number of births was 1 (range: 0–8).This study divided the age into 5 age groups ≤ 30 (n = 654, 18.91%), 31–40 (n = 1258, 36.37%), 41–50 (n = 1011, 29.23%), 51–60 (n = 448, 12.95%), and > 60 (n = 88, 2.54%).

### Distribution of histological results among different genotypes in ASC-US women

**Fig. 2:**
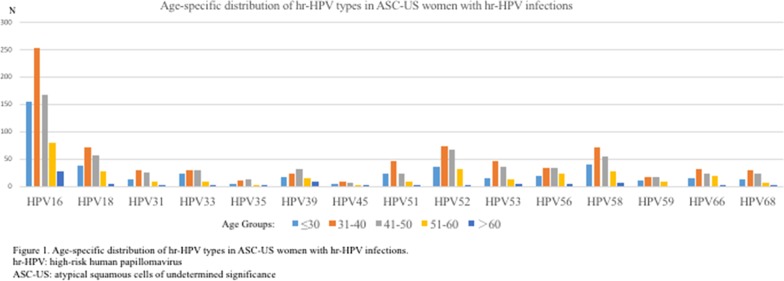
Age-specific distribution of hr-HPV types in ASC-US women with hr-HPV infections. hr-HPV: high-risk human papillomavirus, ASC-US: atypical squamous cells of undetermined significance

The common hr-HPV genotype was shown in Table 1 and Fig. 2, in women with ASC-US.

**Table 1:** Distribution of hr-HPV genotypes in hr-HPV-positive/ASC-US women

Age	hr-HPV types															N^a^
	16	18	31	33	35	39	45	51	52	53	56	58	59	66	68	
≤ 30	155	37	13	22	4	16	4	22	36	15	18	39	10	14	12	654
31–40	253	70	29	29	11	23	9	46	72	46	33	70	17	31	29	1258
41–50	167	56	26	29	13	32	7	22	66	36	34	55	16	22	23	1011
51–60	80	27	8	9	1	15	1	9	32	12	24	27	9	18	6	448
> 60	28	4	1	2	1	8	1	2	2	5	5	7	0	2	1	88
N^b^	683	194	77	91	30	86	22	101	209	114	114	198	52	87	71	3459

ASC-US, atypical squamous cells of undetermined significance; hr-HPV, high-risk human papillomavirus

^a^The total number of people with hr-HPV positive in the corresponding age groups and this table did not list all HPV infection types

^b^The sum of women with the type-specific hr-HPV infection(s) and it would be counted multiple times if the person has multiple hr-HPV infections

The HSIL+ detection rates were different in different hr-HPV genotypes, and the rates of HSIL+ ranged from 8.51 to 63.09% in ASC-US women with single hr-HPV infection. The HSIL+ detection rate of women with single HPV16 infection (63.09%) was the highest, followed by HPV33 (57.50%), HPV51 (36.11%), HPV58 (36.11%), HPV52 (28.28%), HPV18 (26.37%), HPV66 (19.35%), HPV39 (18.92%), HPV53 (15.00%), and HPV56 (8.51%). And, other hr-HPV genotypes (except HPV 16, 18, 33, 39, 51, 52, 53, 56, 58, and 66) were grouped into the group of "others" due to the small number of people included in women with the single hr-HPV infection. The HSIL+ detection rate in the "others" group was 30.09% (Table 2). Overall, a total of 1403 women had single HPV infection, of which 414 (29.51%) were diagnosed as HSIL and 22 (1.57%) women were diagnosed as cervical cancer by histological test.

**Table 2:** Distribution of oncogenic hr-HPV types and histological results among hr-HPV-positive/ASC-US women

HPV types	Histological results					
	Total	Normal (n, %)	LSIL (n, %)	HSIL (n, %)	CA (n, %)	HSIL+ (n,%)
HPV16	401	97 (24.19)	51 (12.72)	234 (58.35)	19 (4.74)	253 (63.09)
HPV18	91	42 (46.15)	25 (27.27)	21 (23.08)	3 (3.30)	24 (26.37)
HPV33	40	11 (27.50)	6 (15.00)	22 (55.00)	1 (2.50)	23 (57.50)
HPV39	37	17 (45.95)	13 (35.14)	7 (18.92)	0 (0.00)	7 (18.92)
HPV51	36	16 (44.44)	7 (19.44)	13 (36.11)	0 (0.00)	13 (36.11)
HPV52	99	48 (48.48)	23 (23.23)	28 (28.28)	0 (0.00)	28 (28.28)
HPV53	40	29 (72.50)	5 (12.50)	6 (15.00)	0 (0.00)	6 (15.00)
HPV56	47	33 (70.21)	10 (21.28)	4 (8.51)	0 (0.00)	4 (8.51)
HPV58	108	38 (35.19)	31 (28.70)	39 (36.11)	0 (0.00)	39 (36.11)
HPV66	31	16 (51.61)	9 (29.03)	6 (19.35)	0 (0.00)	6 (19.35)
Other^a^	113	49 (43.36)	30 (26.55)	34 (30.09)	0 (0.00)	34 (30.09)
2 types^b^	440	161 (36.59)	104 (23.64)	170 (38.64)	5 (1.14)	175 (39.77)
3 types^c^	94	34 (36.17)	21 (22.34)	39 (41.49)	0 (0.00)	39 (41.49)
≥ 4 types^d^	20	6 (30.00)	5 (25.00)	9 (45.00)	0 (0.00)	9 (45.00)
12 hr-HPV^e^	376	179 (47.61)	75 (19.95)	121 (32.18)	1 (0.27)	122 (32.45)
HC2	1245	637 (51.16)	250 (23.24)	347 (27.87)	11 (0.88)	358 (28.76)
ANY^f^	241	92 (38.17)	56 (23.24)	88 (36.51)	5 (2.07)	93 (38.59)
N	3459	1505 (43.51)	721 (20.84)	1188 (34.35)	45 (1.30)	1233 (35.65)

ASC-US, atypical squamous cells of undetermined significance; HPV, human papillomavirus; hr-HPV, high-risk human papillomavirus; LSIL, low-grade squamous intraepithelial lesion; HSIL, high-grade squamous intraepithelial lesion; CA, cervical cancer; HSIL+, high-grade squamous intraepithelial lesion or worse

^a^Single hr-HPV infection except HPV16, 18, 33, 39, 51, 52, 53, 56, 58 and 66

^b^Double infections with hr-HPV

^c^Triple infections

^d^4 or more than kinds hr-HPV infections

^e^Infection(s) with a pool of 12 other hr-HPV types (including HPV 31,33,35,39,45,51,52,56,58,59,66 and 68) that did not know the specific type of HPV (from Cobas 4800 HPV test)

^f^hr-HPV infection that did not know the specific type of HPV (from Cervista HPV HR (Hologic) or Hybrid Capture 2 test)

Among women with the multiple infections, the HSIL+ detection rates in women with double hr-HPV infections, triple hr-HPV infections, and quadruple or more hr-HPV infections were 39.77%, 41.49%, and 45.00%, respectively (Table 2).

A total of 45 (1.30%) of the women in this study were diagnosed with cervical cancer, including 24 women with HPV16 infections (including 19 women with HPV16 single infection and 5 women with HPV16 multiple infections), 3 women with HPV18 single infection, 1 woman with HPV33 single infection, 1 woman with 12 hr-HPV infection (from Cobas 4800 HPV test), and 16 women with unidentified hr-HPV genotypes infections (11 women were HC2 positive, and 5 women belonged to ANY group).

### Distribution of histological results among viral load groups in ASC-US women

1245 women tested positive for hr-HPV by HC2 test. 42.93% (n = 398) of women with ASC-US had normal histological results, 22.76% (n = 211) had LSIL, 33.23% (n = 308) had HSIL, and 1.08% (n = 308) had cancer. 34.30% (n = 318) of women were diagnosed with HSIL+. According to the viral load of hr-HPV, women were divided into 3 groups: low viral load group (1 ≤ RLU/CO < 10, n = 189), medium viral load group (10 ≤ RLU/CO < 100, n = 212), and high viral load group (RLU/CO ≥ 100, n = 526). The pathological distribution of these three viral-load groups was shown in Table 3. The percentage of women with normal pathology in the low-viral-load group (65.61%) was highest, followed by intermediate-viral-load group (41.98%) and high-viral-load group (35.17%). However, detection rate of HSIL+ in low-viral-load group was lowest, followed by intermediate-viral-load group (34.91%) and high-viral-load group (40.68%), and there was is statistically significant between detection rate of HSIL+ and hr-HPV viral load (χ^2^ = 35.03, *p* < 0.0001).

**Table 3:** Distribution of histological results among viral load groups in ASC-US women

Viral load	Histological results					
	Total	Normal (n, %)	LSIL (n, %)	HSIL (n, %)	CA (n,%)	HSIL+ (n, %)
1 ≤ RLU/CO < 10	189	124 (65.61%)	35 (18.52%)	28 (14.81%)	2 (1.06%)	30 (15.87%)
10 ≤ RLU/CO < 100	212	89 (41.98%)	49 (23.11%)	72 (33.96%)	2 (0.94%)	74 (34.91%)
RLU/CO ≥ 100	526	185 (35.17%)	127 (24.14%)	208 (39.54%)	6 (1.14%)	214 (40.68%)
N	927	398 (42.93%)	211 (22.76%)	308 (33.23%)	10 (1.08%)	318 (34.30%)

ASC-US, atypical squamous cells of undetermined significance; RLU/CO, the ratio relative-light-units /cut-off; LSIL, low-grade squamous intraepithelial lesion; HSIL, high-grade squamous intraepithelial lesion; CA, cervical cancer; HSIL+, high-grade squamous intraepithelial lesion and cervical cancer

### Distribution of histological results among age groups in ASC-US women

**Fig. 3:**
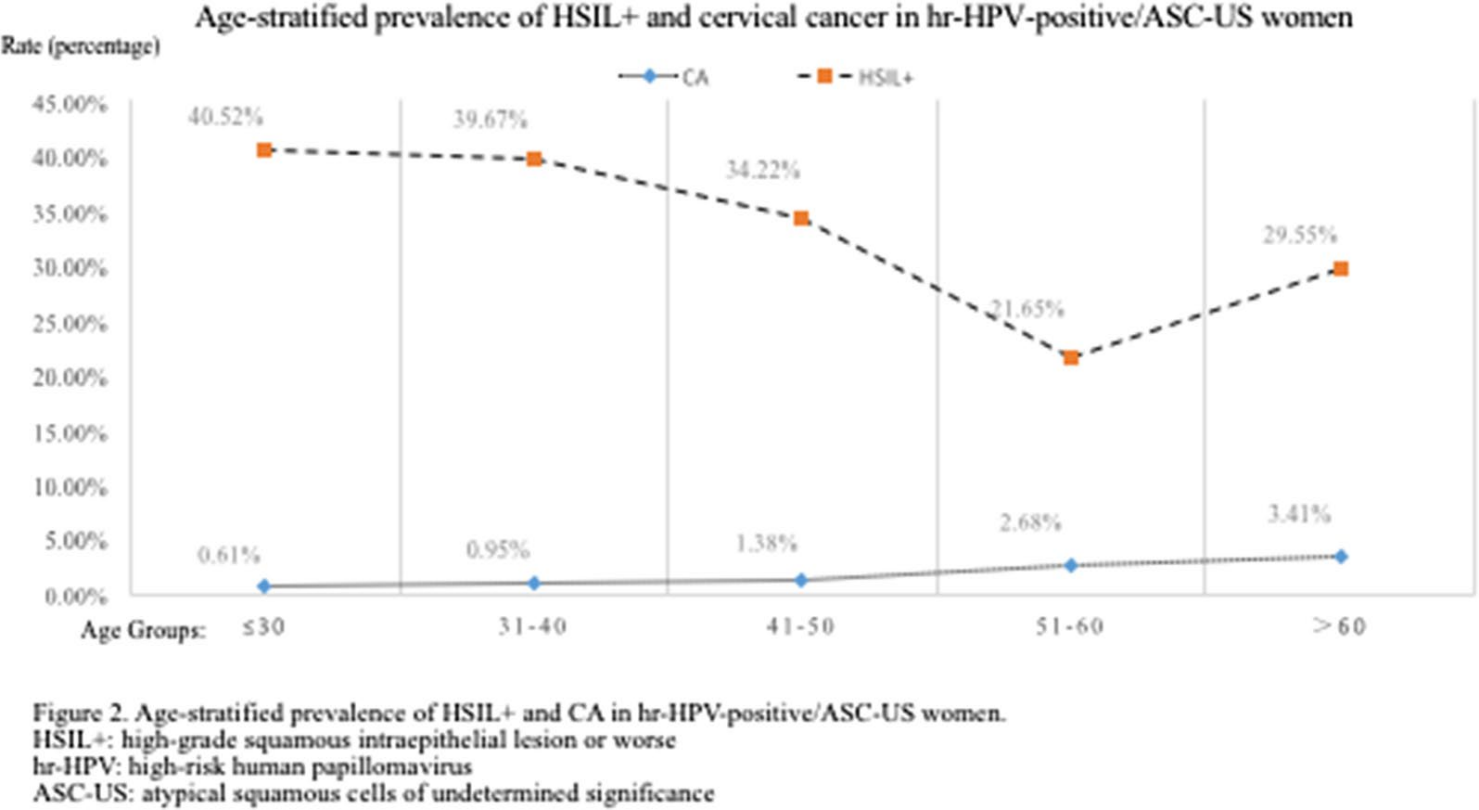
Age-stratified prevalence of HSIL+ and CA in hr-HPV-positive/ASC-US women. HSIL+: high-grade squamous intraepithelial lesion or worse, hr-HPV: high-risk human papillomavirus, ASC-US: atypical squamous cells of undetermined significance

Age-stratified prevalence of HSIL+ and cervical cancer in hr-HPV-positive/ASC-US women is shown in Fig. 3. The HSIL+ detection rate of ≤ 30-year-old age group (40.52%) was the highest; however, the detection rate of HSIL+ (21.65%) was the lowest in the 51–60-year-old group. The women in ≤ 30 (OR = 2.465; 95% CI 1.875–3.241), 31–40 (OR = 2.379; 95% CI 1.850–3.060), 41–50 (OR = 1.883; 95% CI 1.452–2.441) year-old groups had significantly higher risk of HSIL+ compared with those in 51–60-year-old group (OR regarded as 1.00). There was no statistically significant increase in the rate detection of HSIL+ in the group of > 60 years by 36.49% (29.55% vs 21.65%; OR = 1.517; 95% CI 0.911–2.527), compared with the 51–60-year-old group (Table 4).

**Table 4:** Distribution of histological results among viral load groups in ASC-US women

Age	Histological results							
	Total	Normal (n, %)	LSIL (n, %)	HSIL (n,%)	CA (n,%)	CA:OR (95% CI)	HSIL+ (n,%)	HSIL+:OR (95% CI)
≤ 30	654	253 (38.69%)	136 (20.80%)	261 (39.91%)	4 (0.61%)	1.00	265 (40.52%)	2.465 (1.875–3.241)
31–40	1258	500 (39.75%)	259 (20.59%)	487 (38.71%)	12 (0.95%)	1.565 (0.503–4.872)	499 (39.67%)	2.379 (1.850–3.060)
41–50	1011	464 (45.90%)	201 (19.77%)	332 (32.84%)	14 (1.38%)	2.282 (0.748–6.936)	346 (34.22%)	1.883 (1.452–2.441)
51–60	448	240 (53.57%)	110 (24.55%)	85 (18.97%)	12 (2.68%)	4.472 (1.433–13.957)	97 (21.65%)	1.00
> 60	88	48 (54.55%)	15 (17.05%)	23 (26.14%)	3 (3.41%)	5.735 (1.262–26.064)	26 (29.55%)	1.517 (0.911–2.527)
N	3459	1505 (43.51%)	721 (20.84%)	1188 (34.35%)	45 (1.30%)	–	1233 (35.65%)	–

ASC-US, atypical squamous cells of undetermined significance; HPV, human papillomavirus; hr-HPV, high-risk human papillomavirus; LSIL, low-grade squamous intraepithelial lesion; HSIL, high-grade squamous intraepithelial lesion; CA, cervical cancer; HSIL+, high-grade squamous intraepithelial lesion and cervical cancer; OR, odds ratio; CI, confidence interval

The cervical cancer detection rates were 0.61%, 0.95%, 1.38%, 2.68% and 3.41%, respectively, in group of women aged ≤ 30 years, 31–40 years, 41–50 years, 51–60 years and > 60 years. When regarding the rate of cervical cancer of women in ≤ 30-year-old group who had the lowest rate of cervical cancer as the compared baseline (OR as 1.00), the women in ≤ 51–60 (OR = 4.472; 95% CI 1.433–13.957) and > 60 (OR = 5.735; 95% CI 1.262–26.064) year-old groups were at increased risk for cervical cancer. There was no statistically significant increase in the rates detection of cervical cancer in the group of 31–40 years by 55.74% (0.95% vs 0.61%; OR = 1.565; 95% CI 0.503–4.872) and the group of 41–50 years by 126.23% (1.38% vs 0.61%; OR = 2.282; 95% CI 0.748–6.963), compared with the ≤ 30-year-old group (Table 4).

## Discussion

Currently, the HPV vaccine is gradually being used in the developing countries, but it has not fully covered all the regions. The effect of the HPV vaccination will reveal after several decades. In addition, the cervical cancer screening is still an essential method to prevent and treat cervical cancer for older women. Therefore, it’s significant to adopt the cervical cancer screening method to diagnose and prevent the cervical cancer.

ASC-US is an ambiguous term and an exclusionary diagnosis that suggests a risk of disease rather than a definitive diagnosis of abnormal lesions. For women with ASC-US, if the diagnosis is not clear or not timely, the optimal treatment will be delayed; on the other hand, the excessive diagnosis and treatment will not only bring physical and psychological burden to the women, but also can cause adverse pregnancy outcomes [14, 15]. In addition, it will increase the family and society economic burden and will not allocate medical resources reasonably.

This retrospective cross-sectional single-center study in China, lasting for 7 years, collected clinical data of 250,000 women who underwent HPV and TCT test at Qilu Hospital of Shandong University. The frequency of ASC-US was 2.80% (n = 7001) in cervical screenings of this center, which was suggested the center strictly controlled the frequency of occurrence of ASC-US and reduced the phenomenon of ascus as the “Garbage Dump”, according previous research [13]. Some studies have reported that the prevalence of CIN2+ among women with ASC-US is between 5% -39% [16–18]. The detection rate of HSIL+ in hr-HPV-positive/ASC-US women in this study was 35.65%, which was higher than some studies. The possible reasons are as follows: (1) The target population of this study was hr-HPV-positive/ASC-US women, while the target population of other studies were ASC-US women (including hr-HPV positive and negative women). The reported prevalence of hr-HPV among women with ASC-US in most studies was 23% to 74% [19]. (2) This study was a retrospective study with the possibility of incomplete data collection.

In this cross-sectional study, the rates of HSIL+ ranged from 8.51% of HPV 56 to 63.09% of HPV16 in ASC-US women with single hr-HPV infection. The HSIL+ detection rates in women with HPV multiple infections was more than 39.77%. Previous research reported HPV-positive/ ASC-US women would have similar the 2-year cumulative risk of HSIL+ and be clinically equivalent with women with LSIL Pap results. We look forward to relevant longitudinal researches on relationship between HPV genotypes and risk of cervical lesions.

Systematic review [20, 21] and a large randomized trial [11] consistently showed that, compared with repeat cytology, the accuracy of HC2 to detect underlying HSIL+ was higher in triage of women with ASC-US cytology. Previous studies suggested that the test may optimized by using a cutoff higher than this 1.0 pg/mL [22, 23]. The rates of HSIL+ in higher viral road of women with ASC-US cytology were higher (Cochran-Armitage Trend test χ^2^ = 35.03, *p* < 0.0001) in this study.

The highest detection rate of HSIL+ (40.52%) was in the age group of women aged ≤ 30 years, and the lowest detection rate of HSIL+ (21.65%) was in the 51–60-year-old group in this study. A large longitudinal cohort study lasting 7 years within the Guanacaste population showed older women had a similar or slightly decreased risk of HSIL+ and especially CIN 3 compared with younger women [24]. In addition, a systematic review included in 103 studies (including more than 12,400,000 women) found that HSIL prevalence in Asia peaked at a relatively younger age (25 to 40 years) [25]. The results in these studies are in coincidence with our findings. When calculating the cervical cancer detection rate, this study showed that the value of the cervical cancer detection rate increased with age, and older women had higher cervical cancer detection rates than younger women. The reasons are as follows:

1. Due to hormone levels, the use of intrauterine device, more sexual behavior and inflammation in younger women, the cells of those with benign morphological changes mixed with cells of true precancerous lesions, and it may interfere doctors’ diagnosis.
2. HPV prevalence rate among younger women are higher, and the rate of HPV prevalence is highly age-related and decreases with age [26]. According to previous study, the HPV prevalence rate could be as high as 70% for women of aged < 25 years [27].
3. The previous research found that CIN1 and a subset CIN2 lesions are clinical manifestations of the result of productive hr-HPV infectio [28], which can regularly regress within 1–2 years spontaneously and have a low risk to progress to invasive carcinom [29].
4. Older women could clear newly HPV infections (including HPV16 infections) as quickly as younger women [24]. So, the lowest detection rate of HSIL+ was in the group aged 51–60 years old.
5. Lower estrogen levels, thinning of the epithelium, decreasing lactobacilli and some genital tract infections combined diabetes etc., create favorable conditions for HPV infection in older women, especially for > 60 years old women.
6. Another subset of CIN2 lesions and CIN3 lesions are clinical manifestations of the result of transforming hr-HPV infection, which is characterized by a dysregulated expression of E6 and E7 viral oncogenes [28]. It would take the long time of 20–30 years for the progression to invasive carcinoma from precancerous lesion in most patients [10].
7. What’s more, persistent hr-HPV infection is a risk factor for cervical cancer and the risk of persistence increases with the increasing age of the woman. Therefore, older ASC-US women have a higher cervical cancer detection rate.

However, this study showed there was no statistically significant increase in the rate detection of HSIL in the group of > 60 years by 36.49% (29.55% vs 21.65%), compared with the 51–60 years group, which may be related to the relatively limited sample we studied.

A 2013 Cochrane Meta-analysis studied triage ASC-US by repeat cytology versus HPV testing and found that similar pooled CIN2+ and CIN3+ specificities that likely will translate to similar overtreatment rates [12]. So, one way to solve the problem of overtreatment is to consider alternatives new method to triage women with ASC-US. Molecular markers may help identify cells with abnormal cell morphology. P16 or dual p16 and Ki-67 immunostaining on cytological preparations provides a promising method to triage HPV-positive women [30, 31]. Biomarkers based on DNA methylation includes methylation markers representing various combinations of genes, such as SRY-box 1 (SOX1), PAX1, NK6 homeobox 1 (NKX6-1) [32], junctional adhesion molecule 3 (JAM3), EPB41L3, TERT, C13ORF18 [33], and CADM1/MAL [34].

What’s more, this study found that women infected with HPV52 and HPV58 were also more common, except HPV16 and HPV18, in China. Therefore, it seems a wise choice to get a vaccine containing multiple hr-HPV types. Currently, there are two-valent HPV vaccines (HPV 16/18), four-valent HPV vaccines (HPV 6/11/16/18), and nine-valent HPV vaccines (HPV 6/11/16/18/31/33/45/52/58). Therefore, women should choose nine-valent HPV vaccines in China, if conditions permit.

Combined with knowledge about the biology of CIN and results of this study, here are a few suggestions to triage women with ASC-US cytology. Firstly, we should ensure the quality of TCT test and strictly control the occurrence frequency of ASC-US. It has been suggested that good management requires that the frequency of ASC be maintained at < 5% of all cervical screenings [11], and ASC:SIL ratio be maintained at < 1.5. Secondly, ASC-US women with obvious inflammation should accept anti-inflammatory treatment, and repeat cytology test 4–6 months after inflammation disappearing, especially young women. If the cytological results of women ≥ ASC-US, then they are recommended to refer to colposcopy. In addition, we should strictly follow up on young women who have been diagnosed as HSIL by histological examination, rather than implementing excessive treatment. Finally, it is more and more important for us to find a new triage test.

## Conclusions

ASC-US women with HPV 16/18/33/51/52/58 single infection and multiple infections, as well as high HPV viral loads, have high risk of HSIL+. In addition, the rate of HSIL+ was higher and the rate of cervical cancer was lower in younger hr-HPV-positive/ ASC-US women, in contrast with older women.

### Limitations

This study was a retrospective single-center study with the possibility of incomplete data collection and limited data sources. What’s more, women enrolled in this study were tested by there HPV tests and different HPV tests varied in sensitivity and specificity. Relatively large prospective studies were expected to confirm the findings of this study in the future.

## Data Availability

We declared that materials described in the manuscript, including all relevant raw data, will be freely available to any scientist wishing to use them for non-commercial purposes, without breaching participant confidentiality.

## Abbreviations

hr-HPV: High-risk human papillomavirus
TCT: Thinprep cytologic test
ASC-US: Atypical squamous cells of undetermined significance
CIN: Cervical intraepithelial neoplasia
HC2: Hybrid capture 2 test
RLU/CO: The ratio relative-light-units /cut-off
LSIL: Low-grade squamous intraepithelial lesion
HSIL: High-grade squamous intraepithelial lesion
HSIL+: High-grade squamous intraepithelial lesion and cervical cancer
